# Conservation Wildflower Plantings Do Not Enhance On-Farm Abundance of *Amblyomma americanum* (Ixodida: Ixodidae)

**DOI:** 10.3390/insects11090617

**Published:** 2020-09-09

**Authors:** Christopher McCullough, Gina Angelella, Megan O’Rourke

**Affiliations:** 1 School of Plant and Environmental Sciences, 306 Saunders Hall, Virginia Tech, Blacksburg, VA 24061, USA; Gina.Angelella@usda.gov (G.A.); megorust@vt.edu (M.O.); 2 Department of Entomology, 216 Price Hall, Virginia Tech, Blacksburg, VA 24061, USA; 3 Agricultural Research Service, Temperate Tree Fruit and Vegetable Research: USDA Unit, 5320 Konnowac Pass Road, Wapato, WA 98951, USA; 4 National Institute of Food and Agriculture: USDA, Kansas City, MO 64133, USA

**Keywords:** pollinator, tick, ecosystem services, vector ecology

## Abstract

**Simple Summary:**

Planting wildflowers is a commonly used tool to conserve pollinators. However, it is possible that wildflower plantings may inadvertently aid tick species, complicating both vector control and pollinator conservation programs. In this study, we tested whether conservation wildflower plantings enhanced the on-farm abundance of the lone star tick, *Amblyomma americanum* (L.). Over two years, *A. americanum* were sampled using dry ice traps in wildflower plots, weedy field margins, and forested areas. We found no more *A. americanum* in wildflower plots than in weedy field margins. Forested areas harbored the greatest number of *A. americanum* sampled. Overall, wildflower plots do not pose an increased risk of exposure to *A. americanum* on farms.

**Abstract:**

Planting wildflowers is a commonly suggested measure to conserve pollinators. While beneficial for pollinators, plots of wildflowers may be inadvertently performing an ecosystem disservice by providing a suitable habitat for arthropod disease vectors like ticks. The lone star tick, *Amblyomma americanum* (L.), is a medically important tick species that might be able to utilize wildflower plantings as a suitable habitat. In this two-year study, ticks were sampled using dry ice baited traps from wildflower plots, weedy field margins, and forested areas to determine if wildflower plantings were increasing the on-farm abundance of *A. americanum*. Abiotic and biotic environmental variables were also measured to better understand which factors affect *A. americanum* abundance. We found no more *A. americanum* in wildflower plots than in weedy field margins. Forested areas harbored the greatest number of *A. americanum* sampled. The height of the vegetation in the sampled habitats was a significant factor in determining *A. americanum* abundance. Depending on the sampled habitat and life stage, this relationship can be positive or negative. The relationship with vegetation height may be related to the behavior of the white-tailed deer and the questing success of *A. americanum*. Overall, wildflower plots do not pose an increased risk of exposure to *A. americanum* on farms.

## 1. Introduction

The lone star tick, *Amblyomma americanum* (L.). (Ixodida: Ixodidae), is an aggressively-biting species that is a nuisance to humans and a pest of livestock [1]. It has been gaining attention as an important vector of human diseases, including erlichiosis, tuleramia, and Heartland virus [2,3]. More recently, *A. americanum* has been implicated in triggering red meat anaphylaxis caused by the sugar galactose-α-1,3-galactose that is injected during feeding [4]. The range of *A. americanum* has been expanding northward from the southeastern United States and is likely to continue moving northward under current climate change conditions [5,6]. This expanding range will also expand the range of the diseases vectored by *A. americanum*.

*Amblyomma americanum* is the most abundant tick species sampled in southeastern Virginia [7]. In this region, adult and nymphal *A. americanum* are most active from late April to mid-July, and larvae are active from August to October [8]. It takes three blood meals for *A. americanum* to complete their lifecycle: one to molt from larva to nymph, another to molt from nymph to adult, and the final one to produce eggs. *Amblyomma americanum* utilize a wide variety of hosts like small mammals and birds, but white-tailed deer, *Odocoileus virginianus* (Zimmerman), are considered the primary host [9,10]. *Amblyomma americanum* primarily disperse opportunistically on hosts but have been documented questing up to five meters in response to CO_2_ plumes in mark-recapture studies [11].

*Amblyomma americanum* spend most of their life off-host, subjecting them to abiotic conditions in the environment. They protect themselves against desiccation with cuticular wax deposits to inhibit water loss and can absorb moisture directly from the air [12,13,14]. *Amblyomma americanum* quest during times of the day when temperatures are high and relative humidity is low [15,16], and generally seek environments that experience low temperature variation and have high relative humidity [17]. Forests provide such favorable conditions for *A. americanum* to survive and are generally more preferred habitats than grasslands [18,19]. 

Habitat manipulations can affect the abundance of *A. americanum* by altering host behavior and microclimates. Areas where the invasive shrub Amur honeysuckle, *Lonircera maackii* (Rupr.) Herder, had been removed produced lower *A. americanum* densities than areas with the shrub, due to the preference of white-tailed deer for areas with *L. maackii* [20]. The westward expansion of the eastern red cedar, *Juniperus virginiana* L., in Oklahoma is believed to be facilitating a similar westward expansion of *A. americanum* by providing both better environmental conditions for the tick and white-tailed deer [21].

*Amblyomma americanum* mortality was higher in plots with the invasive Japanese stiltgrass, *Microstegium vimineum* (Trin.), compared to plots without the plant [22]; this was because plots with *M. vimineum* had higher temperatures and lower humidity than control plots [22]. This effect has been observed with another tick species, *Ixodes scapularis* Say. Areas where the invasive shrub Japanese barberry, *Berberis thundbergii* de Candolle, had established had higher daily average relative humidity values at ground level than plots without Japanese barberry, or those where it had been thinned [23]. Subsequently, more *I. scapularis* were sampled from plots with Japanese barberry compared to control plots [23].

Concurrent with concerns about the changes in tick-vectored diseases are concerns about pollinator declines. One of the primary drivers of pollinator decline is habitat loss, in conjunction with pesticide exposure and diseases [24]. One mitigation strategy is the planting of wildflower plots to provide resources for bees, which can increase their abundance and diversity [25]. These plots also provide resources for other beneficial arthropods, such as natural enemies of crop pests [26]. The installation of these plots is subsidized in the United States by government programs such as the Environmental Quality Incentives Program [27]. From 2009 to 2018, this program has helped pay for habitat management that is beneficial for pollinators on over 16,000,000 acres of land [27,28]. 

It is unknown, however, whether wildflower plots provide favorable habitats for ticks and could potentially increase the risk of exposure to people and animals to *A. americanum*. As called for by Ginsberg et al. [29], the impacts of pollinator conservation and vector control on each other need to be researched to minimize potential negative outcomes. Managing wildflower plots requires that they be mowed each year during the dormant season [30]. This annual mowing could potentially build up a duff layer that provides a critical microclimate with high humidity and stable temperatures that would be hospitable for *A. americanum*. Given that the removal of plants can reduce *A. americanum* populations [20,21], does the addition of plants aid *A. americanum* populations? The purpose of this study was to determine if on-farm wildflower plots can serve as quality habitat for *A. americanum*. 

## 2. Materials and Methods

### 2.1. Study Area

Tick surveys were conducted at 10 farms in eastern Virginia and Maryland in 2018, and nine of the same farms in 2019. Nine of these farms had one wildflower plot installed during the spring of 2016; one farm had their wildflower plot seeded in the spring of 2015. Wildflower plot sizes ranged from 561–8600 m^2^, with an average size of 2360 m^2^. Three different wildflower mixes were used that were adapted to local soil conditions (Table 1). Wildflower plot establishment procedures were similar for each mix used. Generally, the site of the wildflower plot was tilled, packed, seeded, and packed once more—see Angelella and O’Rourke for further details of the fields seeded with the well-draining mix [31]. After establishment, the plots were mowed annually during the plant dormant season, between November and March. 

### 2.2. Sampling

**Figure 1:**
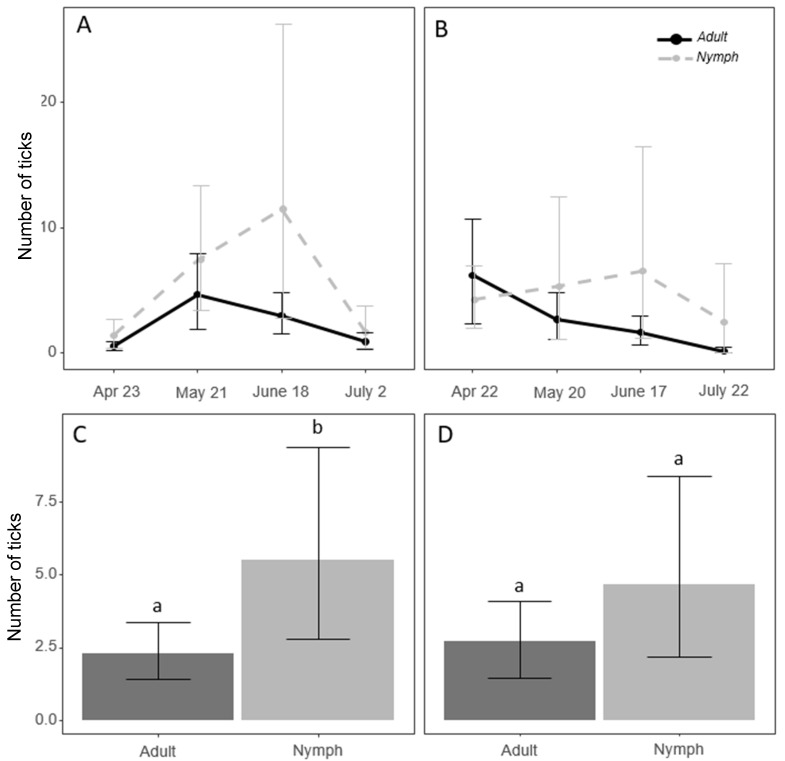
Average number of *Amblyomma americanum* adults and nymphs sampled during each week of sampling in 2018 (**A**) and 2019 (**B**). The yearly average abundance of adults and nymphs sampled in 2018 (**C**) and 2019 (**D**). Means with the same letter are not significantly different. (Tukey’s Honestly Significant Difference) (*p* < 0.05).

Ticks were sampled with dry ice traps [11]. The traps were 8-liter coolers, measuring 33 cm × 24 cm × 22 cm (Igloo Coolers, Katy, TX, USA) with 13 mm diameter holes drilled into each side. The traps were loaded with 2 kg of dry ice. A 5 cm band of Shurtape Indoor/Outdoor tape (Hickory, NC, USA) was placed around the outside of the traps. Three habitats were sampled at each farm: the wildflower plot, a weedy field margin, and a nearby forest. One dry ice trap was placed in each sampling habitat at each location on each sampling date; the traps were placed at least 10 m apart. The traps were set between 9:00 a.m. and 12:00 p.m. and left in the field for 24 h. Each field was sampled once per month from April to July in 2018 and 2019 (Figure 1). All ticks caught were placed in 95% ethanol and later identified by species and separated into nymphs and adults. 

### 2.3. Environmental Variables

Temperature and relative humidity at the soil surface were recorded every half-hour for the full duration of trap deployment in each habitat at each field site during each round of sampling using a Hobo U23 Pro V2 data logger (Onset; Bourne, MA, USA). Vegetation height was measured at five locations during each sampling date. The height of herbaceous vegetation was measured at the trap location and 4 m away from the trap in each cardinal direction [32]. The duff depth was measured as the distance from the bare soil to the top of the organic matter on the ground and was measured after the last round of tick sampling each year at the same locations as the herbaceous vegetation height. 

### 2.4. Statistical Analysis

To determine if wildflower plots increased *A. americanum* abundance relative to weedy field margins, a generalized linear mixed model fit to a negative binomial distribution was used to analyze these data. The interaction of the sampling habitat and tick life stage (nymph or adult) and their main effects were fixed factors. The field and the sampling date nested in field were set as random effects. Tukey’s Honestly Significant Difference (HSD) was used to test for differences among means. The analyses were done in ‘R’ version 3.5.2 [33] using the package ‘glmmADMB’ [34]. The package ‘emmeans’ was used for multiple comparisons [35].

To investigate the effects of the measured environmental variables on tick abundance, a multimodel approach was used. A set of 10 a priori models was created with predictor environmental variables that are commonly associated with *A. americanum*. The variables tested were: habitat, duff depth, average vegetation height, average relative humidity, and temperature standard deviation. Each variable was tested alone, and then the habitat and the other environmental variables were tested together with main effect and interaction terms in the models. An additional intercept-only model was included as a null model. Models were constructed as generalized linear mixed effects models with a negative binomial distribution. Additionally, the field was included as a random effect, along with the year and sampling date nested within field. Models within four Akaike Information Criterion, adjusted for small sample size, points (AICc) of the top model were selected and averaged [36] using the package ‘MuMIn’ [37]. To reduce the likelihood of including uninformative parameters, the changes in AICc values were compared relative to changes in log likelihood values [38]. All analyses were done separately for nymphs and adults as they can have different responses to the environment [39]. 

### 2.5. Data Availability

These data are available at the VtechData Repository (https://doi.org/10.7294/NFSK-2M11).

## 3. Results

We collected 1165 nymphs and 566 adult *A. americanum* over the two years of sampling. *Amblyomma americanum* nymph abundance peaked in June of both years (Figure 1). A peak in adult abundance was seen in May in 2018 and April in 2019 (Figure 1). On average, more nymphs than adults were detected in 2018 (*z* = 2.91, *p* = 0.003). This effect was not statistically significant in 2019 (*z* = 1.85, *p* = 0.06) (Figure 1).

**Figure 2:**
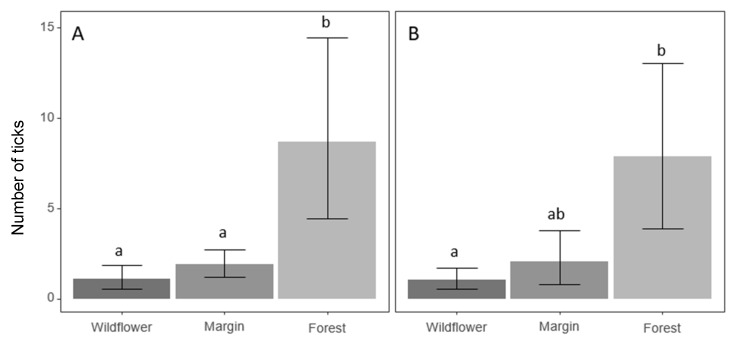
Average number of *A. americanum* sampled from each sampling habit in 2018 (**A**) and 2019 (**B**). Means with the same letter are not significantly different. (Tukey’s Honestly Significant Difference) (*p* < 0.05).

Of the total 1731 *A. americanum* detected, 164 were taken from wildflower plots, 302 from weedy field margins, and 1265 from forest locations. No interaction between life stage and habitat was detected in either year of study. There was no difference in *A. americanum* abundance between wildflower plots and weedy field margins in 2018 and 2019 (Figure 2). *Amblyomma americanum* were detected most often in the forest plots (Figure 2), with significantly fewer *A. americanum* collected in wildflower plots than forest plots in both years (*z* = −5.23, *p* < 0.001; *z* = −3.89, *p* = 0.003). Forests consistently had the thickest duff layers, shortest vegetation, and most stable temperatures of the three habitat types sampled (Table 2).

**Figure 3:**
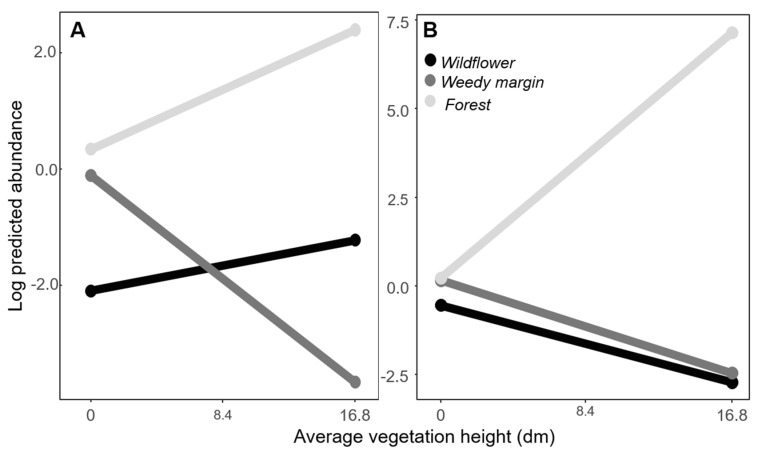
Prediction lines for the abundance of *Ambloymma americanum* adults (**A**) and nymphs (**B**) by sampling habitat in response to vegetation height.

The model with the interaction of habitat type and vegetation height was the top ranked model for predicting adult *A. americanum* abundance (Table 3). The models containing habitat only and the interaction between habitat and duff depth were within 4 AICc points of the top model and were included in model averaging (Table 3). After model averaging, no effects were detected for duff depth (Table 4). Vegetation height had a significant different effect in weedy field margins compared to the other habitats. As the height of vegetation increased in weedy field margins, the abundance of *A. americanum* decreased (*z* = 2.4, *p* = 0.02) (Table 4, Figure 3). 

Similar to adult *A. americanum*, the habitat by vegetation height model was the best predictor of nymph abundance. All other models were more than 4 AICc points higher (Table 5). As vegetation height increased in the forest samples, so did *A. americanum* nymph abundance (*z* = 3.4, *p* = 0.001) (Table 6). This contrasts to wildflower plots and weedy field margins, where nymph abundance decreased with increasing vegetation height (*z* = −2.08, *p* = 0.04) (Table 6, Figure 3).

## 4. Discussion

To our knowledge, this is the first study investigating the interaction of wildflower plots and tick abundance. In this study, wildflower plots planted for pollinator conservation did not inadvertently constitute an ecosystem disservice by simultaneously increasing *A. americanum* abundance. While *A. americanum* were detected in wildflower plots, this habitat harbored fewer of them than weedy field margins. Therefore, wildflower plots do not pose a risk of augmenting on-farm *A. americanum* abundance. 

Vegetation height is playing a role in the differences in adult *A. americanum* abundance between the sampling habitats. These changes could be related to the questing success of *A. americanum* adults on taller vegetation. Adult *A. americanum* could have higher success rates in finding a host in weedy field margins compared to the other habitats sampled. With taller vegetation, adult *A. americanum* would have more area to utilize for questing to attach to larger hosts like white-tailed deer. Many of the weedy field margins sampled were a transition zone from agricultural areas to forested ones. These transition areas are frequented by white-tailed deer as they move from areas of cover to open areas as part of their diurnal movement [40]. Adult *A. americanum* could be investing more in vertical movement in the habitat than horizontal since hosts are not likely to linger. This behavior was seen with *I. scapularis* in habitats that were deemed difficult for ticks to traverse due to the vertical habitat structure [41] With greater success in finding hosts, fewer *A. americanum* would be available to sample. 

*Amblyomma americanum* nymph abundance decreased with taller vegetation in wildflower plots and weedy field margins but increased with taller vegetation in forested areas. Decreases in nymph abundance could be following a similar pattern as the adults; as vegetation height increased, so did questing success. The increase of *A. americanum* nymph abundance with increasing vegetation height in forested areas could be explained by the preference of white-tailed deer to use dense vegetation in forests for bedding sites [40]. With female white-tailed deer having a strong preference for their home range, they could be frequenting bedding areas and dropping engorged larvae. This could create nymphal hotspots in these areas. When the deer return to bed, *A. americanum* nymphs may have the time to successfully attach to the host. *Ixodes scupalris* more actively quest towards a host when the host is stationary [41]. Larger wildflower plots could potentially provide enough cover for deer to use as bedding sites, creating a similar situation. However, given the smaller size of the plots used in this study, this was unlikely to be occurring. A study with *Peromyscus* spp. mice found that 64% of the nests surveyed had *I. scapularis* present, and 87% of all larval ticks present had taken a blood meal [42]. Future studies could examine if specific sites where hosts remain immobile and which they frequently visit are attractive for immature stages of ticks. 

In this study, duff depth was selected as a factor for adult *A. americanum* but was not significant with model averaging. A similar result was seen in Missouri, where duff layer depth was selected as a factor, but not a significant factor in determining adult *A. americanum* abundance [39]. However, duff depth is an important environmental factor for the survival of adult *A. americanum* as it can create a critical microclimate for preventing desiccation. *Amblyomma americanum* adults are sensitive to moisture loss and will seek out moist microclimates after losing only 10–15% of their body weight to counteract desiccation [43]. While the duff layer is important, its presence may be all that matters. In a previous study, *A. americanum* were collected from areas that had shallower duff layers than *I. scapularis,* but never from areas that had no duff layer [44]. Better quantification of the microclimate of the duff layer may also help detect its effects on *A. americanum* abundance, as the duff layer can be 2–3 °C cooler than the ambient air temperature [16]. 

The result that wildflower plots are not increasing on-farm *A. americanum* abundance is encouraging for both pollinator conservation and vector control. Further studies are needed to verify the results in other geographic areas and with different mixes of wildflowers. The mix of wildflower species in a pollinator habitat may have a large influence on the behaviors of tick hosts. A study in Florida observed that white-tailed deer browsed on all 11 wildflower species used in their pollinator mixes [45]. Of the species tested by Degroote et al. [45], two flower species were also present in this study: *Coreopsis lanceolata* L. and *Rudbeckia hirta* L. However, DeGroote et al. [45] found that these were the least and fifth least browsed wildflower species, respectively. If wildflower mixes have species that are attractive to white-tailed deer, tick abundance may likely increase. However, from this study, it is not known if the presence of *A.* *americanum* in the plots indicates that they can complete their lifecycle in this habitat, or if they are simply dropping off of their host. This could include sampling for hosts from within the different habitats to see what role they are playing in driving the difference in *A.* *americanum* abundance. 

Different tick species may have varied levels of attraction to the same habitat. Within a 1-ha forested plot, *A. americanum* and *Ixodes scapularis* were distributed between two different sets of habitat conditions related to each species’ tolerance to desiccation [44]. *Amblyomma americanum* was found in areas with a more open canopy and less shrubby understory compared to *I. scapularis* [44]. If dense stands of ground cover develop within wildflower plots, they could attract rodent hosts of *I. scapularis* [46], potentially increasing *I. scapularis* abundance within its range. This could be more problematic if conservation efforts are focused on the use of longer-lived woody plants, or if herbaceous wildflowers are not mowed regularly. 

## 5. Conclusions

In summary, *A. americanum* were sampled from within wildflower plots, but they are not increasing the risk of exposure to *A. americanum* relative to weedy field margins. The role of hosts in moving *A. americanum* into the wildflower plots is an important factor in the success of *A. americanum* in colonizing these habitats which deserves further study. Understanding how the hosts of *A. americanum* utilize wildflower plots for cover and their preferences for different wildflower species as food could further inform the risks posed by wildflower plots. 

## Figures and Tables

**Table 1:** Wildflower species used in well-draining soils (WD), well-draining replacement (WDr), and poorly draining soils (PD) seed mixes. For WD, WDr, and PD, n = 7, 1, and 2, respectively.

Common Name	Scientific Name	Mix Used in
Narrowleaf mountain mint	*Pycnanthemum tenuifolium*	WD
Plains coreopsis	*Coreopsis tinctoria*	WD, WDr, PD
Partridge pea	*Chamaecrista fasciculata*	WD, WDr, PD
Black-eyed Susan	*Rudbeckia hirta*	WD, WDr
Bergamot, spotted	*Monarda fistulosa*	WD
Lanceleaf coreopsis	*Coreopsis lanceolata*	WD, WDr
Maximilian sunflower	*Helianthus maximilianii*	WD, WDr
Indian blanket	*Gaillardia pulchella*	WD, WDr
Purple coneflower	*Echinacea purpurea*	WD
Spotted beebalm	*Monarda punctate*	WDr
Tickseed sunflower	*Bidens aristosa*	WDr
Showy evening primrose	*Oenothera speciosa*	WDr
Purple-stemmed aster	*Symphyotrichum puniceum var. puniceum*	PD
Common sneezeweed	*Helenium autumnale*	PD
Wrinkleleaf goldenrod	*Solidago rugosa*	PD
Spotted Joe Pye weed	*Eupatoriadelphus fistulosus*	PD
Rattlesnake master	*Eryngium yuccifolium*	PD
Rosemallow	*Hibiscus moscheutos*	PD
Narrowleaf sunflower	*Helianthus angustifolius*	PD

**Table 2:** Yearly averages (mean ± std. error) of environmental factors measured at each sampling location.

Year	Habitat	Vegetation Height (dm)	Duff Depth (cm)	RH	Temp. Standard Deviation (°C)
2018	Wildflower	4.5 ± 0.5	0.62 ± 0.1	86.1 ± 1.0	5.8 ± 0.3
	Weedy margin	4.2 ± 0.4	1.56 ± 0.1	85.4 ± 1.1	6.1 ± 0.3
	Forest	2.2 ± 0.4	3.38 ± 0.2	85.7 ± 1.3	3.4 ± 0.2
2019	Wildflower	6.7 ± 0.6	1.09 ± 0.1	84.5 ± 1.1	6.3 ± 0.4
	Weedy margin	5.1 ± 0.5	1.27 ± 0.1	85.1 ± 1.0	6.3 ± 0.4
	Forest	1.2 ± 0.1	3.87 ± 0.1	82.2 ± 1.4	3.7 ± 0.2

**Table 3:** Model selection for environmental variables affecting the abundance of *A. americanum* adults. Models with interaction terms include the main effects.

Model	Number of Parameters (k)	Log Likelihood (logLik)	Akaike Information Criterion, Small Sample (AICc)	Change in AICc (Δ AICc)	Weight
Habitat × Vegetation Height	10	−346.94	714.88	-	0.539 †
Habitat	7	−350.89	716.29	1.407	0.266 †
Habitat × Duff Depth	10	−348.75	718.51	3.628	0.088 †
Duff depth	6	−353.46	719.3	4.416	0.059
Habitat × Temperature Standard Deviation	10	−349.91	720.83	5.944	0.028
Habitat × Relative Humidity	10	−350.21	721.44	6.552	0.02
Vegetation Height	6	−373.38	759.13	44.25	0
Temperature Standard Deviation	6	−377	766.39	51.504	0
Relative Humidity	6	−382.34	777.06	62.172	0
Intercept only	5	−383.59	777.45	62.564	0

† model selected for averaging; k is the number of parameters in the model.

**Table 4:** Parameter estimates of model averaging using selected models (habitat, habitat × vegetation height, and habitat × duff depth) of environmental variables affecting the abundance of *A. americanum* adults.

Term	Estimate	Adjusted SE	*z* Value	*p* > *z*
Intercept	−1.93	0.64	3.00	0.003 *
Weedy Margin	1.48	0.83	1.78	0.08
Forest	2.25	0.69	3.28	0.001 *
Vegetation Height	0.05	0.07	0.80	0.42
Vegetation Height × Weedy Margin	−0.26	0.11	2.35	0.019 *
Vegetation Height × Forest	0.07	0.14	0.49	0.62
Duff depth	−0.26	0.42	0.63	0.53
Duff depth × Weedy Margin	0.63	0.55	1.16	0.25
Duff depth × Forest	0.61	0.47	1.29	0.20

* significant at *p* < 0.05.

**Table 5:** Model selection for environmental variables affecting the abundance of *A. americanum* nymphs. Models with interaction terms also include the main effects.

Model	k	logLik	AICc	Δ AICc	Weight
Habitat × Vegetation Height	10	−422.78	866.57	-	0.985 †
Habitat	7	−430.55	875.6	9.031	0.011
Habitat × Duff Depth	10	−429.32	879.65	13.078	0.001
Habitat × Relative Humidity	10	−429.41	879.83	13.258	0.001
Habitat × Temperature SD	10	−429.42	879.84	13.272	0.001
Duff depth	6	−436.94	886.26	19.688	0
Vegetation Height	6	−445.33	903.03	36.46	0
Temperature SD	6	−448.95	910.29	43.718	0
Relative Humidity	6	−452.4	917.18	50.612	0
Intercept only	5	−454.4	919.07	52.502	0

† model selected; k is the number of parameters in the model.

**Table 6:** Parameter estimates for the model used to analyze the effects of habitat and vegetation height on the abundance of *A. americanum* nymphs.

Term	Estimate	Std. Error	*z* Value	*p* > *z*
Intercept	−0.55	0.77	−0.71	0.48
Weedy Margin	0.70	0.58	1.22	0.22
Forest	0.77	0.48	1.61	0.11
Vegetation Height	−0.13	0.06	−2.08	0.04 *
Vegetation Height × Weedy Margin	−0.03	0.11	−0.23	0.82
Vegetation Height × Forest	0.54	0.16	3.37	0.001 *

* significant at *p* < 0.05.
